# MapMySmoke: feasibility of a new quit cigarette smoking mobile phone application using integrated geo-positioning technology, and motivational messaging within a primary care setting

**DOI:** 10.1186/s40814-017-0165-4

**Published:** 2017-07-14

**Authors:** Robert S. Schick, Thomas W. Kelsey, John Marston, Kay Samson, Gerald W. Humphris

**Affiliations:** 1 School of Mathematics and Statistics, The Observatory, University of St Andrews, Buchanan Gardens, St Andrews, KY16 9LZ UK; 2 School of Computer Science, University of St Andrews, North Haugh, St Andrews, KY16 9SX UK; 3 Dr Kyle and Partners Surgery, 2 Routine Row, Pittenweem, Anstruther KY10 2LG UK; 4 NHS Fife Smoking Cessation Services, Cameron Hospital, Leven, KY8 5RR UK; 5 School of Medicine, Medical and Biological Sciences, University of St Andrews, North Haugh, St Andrews, KY16 9TF UK; 6 Marine Geospatial Ecology Lab, Nicholas School of the Environment, Duke University, Durham, NC 27708 USA

**Keywords:** Smoking cessation, Smartphone, App, Hidden Markov models, INLA, Geospatial

## Abstract

**Background:**

Approximately 11,000 people die in Scotland each year as a result of smoking-related causes. Quitting smoking is relatively easy; maintaining a quit attempt is a very difficult task with success rates for unaided quit attempts stubbornly remaining in the single digits. Pharmaceutical treatment can improve these rates by lowering the overall reward factor of nicotine. However, these and related nicotine replacement therapies do not operate on, or address, the spatial and contextual aspects of smoking behaviour. With the ubiquity of smartphones that can log spatial, quantitative and qualitative data related to smoking behaviour, there exists a person-centred clinical opportunity to support smokers attempting to quit by first understanding their smoking behaviour and subsequently sending them dynamic messages to encourage health behaviour change within a situational context.

**Methods:**

We have built a smartphone app—MapMySmoke—that works on Android and iOS platforms. The deployment of this app within a clinical National Health Service (NHS) setting has two distinct phases: (1) a 2-week logging phase where pre-quit patients log all of their smoking and craving events; and (2) a post-quit phase where users receive dynamic support messages and can continue to log craving events, and should they occur, relapse events. Following the initial logging phase, patients consult with their general practitioner (GP) or healthcare provider to review their smoking patterns and to outline a precise, individualised quit attempt plan. Our feasibility study consists of assessment of an initial app version during and after use by eight patients recruited from an NHS Fife GP practice. In addition to evaluation of the app as a potential smoking cessation aid, we have assessed the user experience, technological requirements and security of the data flow.

**Results:**

In an initial feasibility study, we have deployed the app for a small number of patients within one GP practice in NHS Fife. We recruited eight patients within one surgery, four of whom actively logged information about their smoking behaviour. Initial feedback was very positive, and users indicated a willingness to log their craving and smoking events. In addition, two out of three patients who completed follow-up interviews noted that the app helped them reduce the number of cigarettes they smoked per day, while the third indicated that it had helped them quit. The study highlighted the use of pushed notifications as a potential technology for maintaining quit attempts, and the security of collection of data was audited. These initial results influenced the design of a planned second larger study, comprised of 100 patients, the primary objectives of which are to use statistical modelling to identify times and places of probable switches into smoking states, and to target these times with dynamic health behaviour messaging.

**Conclusions:**

While the health benefits of quitting smoking are unequivocal, such behaviour change is very difficult to achieve. Many factors are likely to contribute to maintaining smoking behaviour, yet the precise role of cues derived from the spatial environment remains unclear. The rise of smartphones, therefore, allows clinicians the opportunity to better understand the spatial aspects of smoking behaviour and affords them the opportunity to push targeted individualised health support messages at vulnerable times and places.

**Trial registration:**

ClinicalTrial.gov, NCT02932917.

## Background

Approximately 11,000 people die each year in Scotland as a result of smoking-related causes (statistics taken from the Scottish Public Health Observatory, https://scotpho.nhsnss.scot.nhs.uk/scotpho/homeAction.do, last accessed 4 October 2016). Despite many therapies that have been introduced over the years, maintaining a quit attempt remains a monumentally difficult task, and most people require several quit attempts before they successfully maintain cessation at 1 year. Unaided quit attempts are successful only about 3–6% of the time [1]. Quit attempts that are aided by cessation support and pharmaceutical treatment with nicotine replacement therapy are more likely to be successful [1]. However, even the most effective drug-related therapies, e.g. varenicline, only increase success rate to approximately 24% [2]. Despite this increase in success rate, varenicline and other interventions do not operate on the role of the spatial or built environment on smoking behaviour, i.e. certain cues in the environment can still trigger the craving, even when the drug is being taken. We know from clinical studies on rats that spatial patterns can play a big role in nicotine-seeking behaviour [3], and we know in humans that cue-induced triggers play a big role in smoking behaviour [4]. In addition to these issues, we are at a period in history where more and more people carry sophisticated smartphones with them at all times. These devices are capable of monitoring location, and possibly how mood changes with location. Therefore, a research opportunity exists to investigate the role that spatial context plays on three important health behaviours: (1) individual smoking behaviour; (2) maintaining smoking cessation; and (3) what might trigger smoking relapse. Here, we highlight how we will conduct this research within a clinical setting by using a mobile phone app to help monitor smoking behaviour at specific places and times, and to provide patients smartphone-based support in their efforts to quit smoking.

The rise of mobile phones, and in particular smartphones, offers researchers and health practitioners a unique opportunity to capture a more precise and individualised picture of a patient’s smoking behaviour and related emotions and cognitions through time and across space. In theory, these longitudinal health profiles can be used to better target intervention and to support smoking cessation attempts by communicating to the patient at times and places meaningful to the individual [5].

The use of mobile phones in clinical settings is an emerging research thread and is being applied in a variety of health systems including smoking [5]. A recent Cochrane review [6] highlighted 12 studies using mobile and smartphones in smoking cessation. These 12 studies indicate a significant benefit of mobile phone-based intervention, with a relative effect estimate of 1.67. Specifically, mobile phone intervention increases rates of cessation from 53 per 1000 to ~93 per 1000 [6]. Two more recent studies have also shown (a) benefits of one-way (provider to patient) social interchange [7] and (b) effectiveness of social platforms, e.g. WhatsApp, that accompany cessation studies [8].

Moving beyond smoking cessation, developers have flooded the market with apps designed to help foster wellness and health [9], yet the efficacy of many of these apps remains unproven. For example, many apps log ‘health’ or wellness behaviour and provide generic advice, but too often there is no documented record of clinical significance of the app’s intervention [9]. While the technological developments have made this type of communication easy, the community is still catching up to determine the efficacy of such communication. In terms of the delivery of these messages, it is unclear whether or not the timing and location of the patient matters for overall efficacy. Though it seems logical to harness the power of smartphones and the data they collect, we are unaware of apps that provide both individualised advice at times and places that are meaningful. For example, most of the smoking cessation studies rely on SMS-based messaging, which while clearly successful [10], cannot easily be tied to specific geographic locations. This is changing though; the Q Sense app uses geofencing to provide spatially explicit messaging [11]. Geofencing in this context means that the phone’s GPS system establishes a perimeter around a known location, e.g. ‘Home’. When the user enters this location, each event can be tagged as at home, and/or support messages specific to this location can be triggered. In two additional studies, Reitzel et al. [12] and Mitchell et al. [13] both marry ecological momentary assessments (EMA) data with geospatial data. Finally, while apps have flooded the market, relatively few studies are supported and backed by health behaviour change models [14].

Inspired by recent calls to examine the role of the spatial environment in health behaviour [15], we have built a mobile phone application (app), whose clinical deployment is comprised of two distinct phases. In the first phase, the patient logs their smoking behaviour and craving events for 2 weeks. Following this logging phase, the patient will meet with their health professional to review their behaviour. The goals of this meeting will be twofold: (1) to understand the specifics of the patient’s smoking behaviour; to pinpoint, in time and space, the critical cigarettes; and (2) to use motivational interviewing [16, 17] in order to outline a series of quit-related goals and implementation intentions [18]. The app will also capture spatial information connected with relapse events, which may help inform the role the built environment plays in relapse behaviour. Here, we describe the main functionality of the app, details of the feasibility study, our data security protocol and proposed statistical analysis of the data.

## Methods

### Aim, design and setting

We have received ethical approval from the National Health Service to deploy this app with patients in a clinical setting in two feasibility studies of increasing size (approval granted 26 February 2016; REC Reference: 16/WM/0068; IRAS Project ID: 191816). In this paper, we report on the successful completion of the first of these and the use of the initial results to aid the design of the second feasibility study.

The design and setting for the initial feasibility study was a maximum of 10 patients within one general practice surgery in Fife, UK; patients were recruited and monitored by JM. We chose this small number of patients from this practice so that we could quickly get the app into the hands of actual smokers, and therefore more quickly get feedback on the app. To recruit these first 10 patients, we created and displayed a poster in the practice. Also, JM reached out to individual patients to notify them of the study and ask them if they would be interested in participating. Patients who expressed interest in participating were subsequently consented with a different PI from JM.

**Fig. 1:**
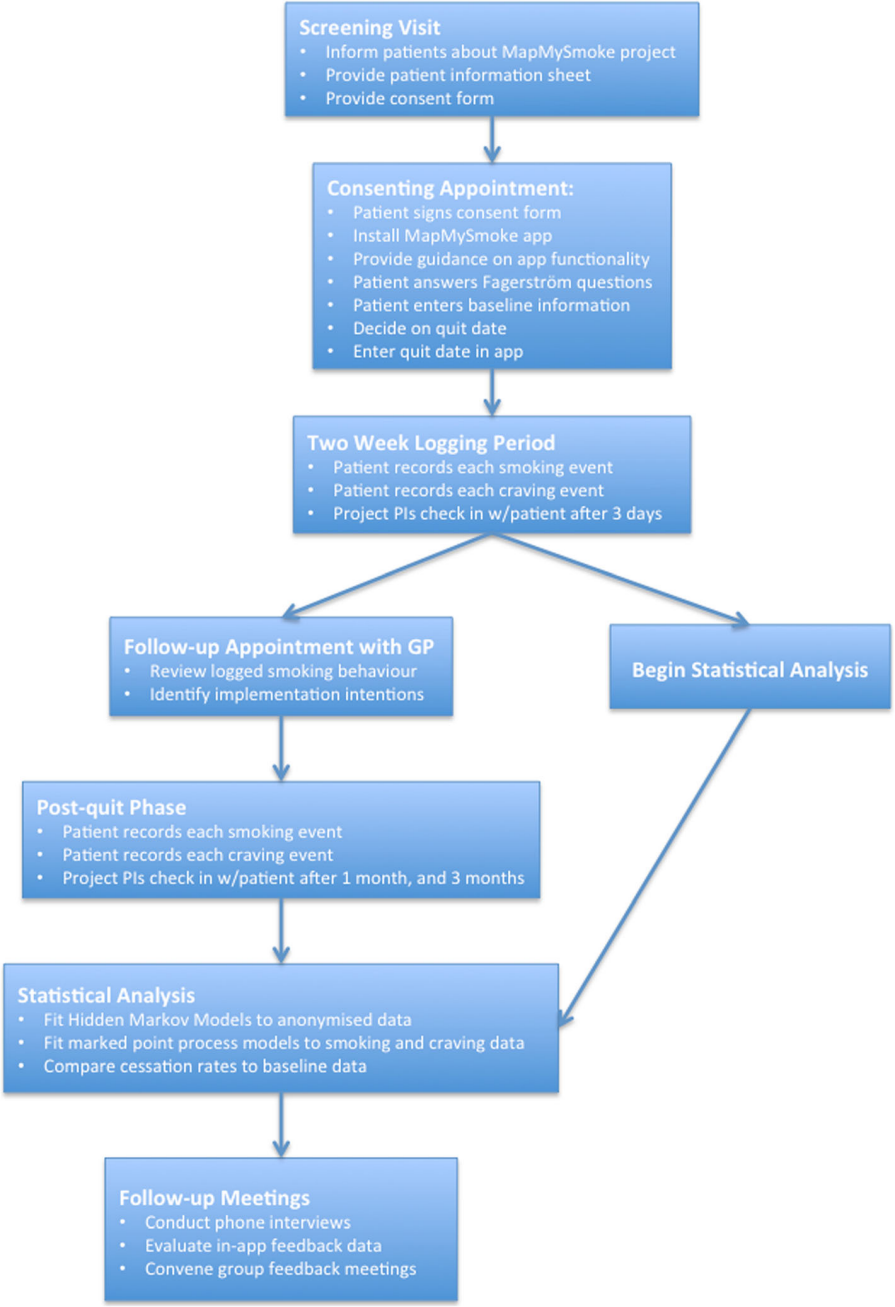
Schematic of patient progress through the MapMySmoke study

The second study will involve up to 100 patients recruited primarily through the NHS Fife Smoking Cessation Services. Target patients for both studies are active smokers interested in quitting, and in possession of a smartphone with a data plan. By active, we mean patients who regularly have one or more cigarettes per week, and specifically that the patients inhale smoke from burning tobacco encased in cigarettes, pipes or cigars. We exclude patients with serious mental illness, and those without a smartphone. The flow of patients through the study is shown in Fig. 1.

### Inclusion criteria

- Live or work in Fife, Scotland, UK
- Age 18 or greater
- Active smoker interested in quitting
- Possess an Android or iOS (at least iOS 7.0) smartphone with a data plan
- Give informed consent

### Exclusion criteria

- Serious mental illness
- Learning difficulty

### Study details

The primary objective of the first small feasibility study was to see if it is possible to deploy the MapMySmoke app within a National Health Service (NHS) setting; that is, we sought to take the app out of the development lab and into the hands of actual patients. In so doing, we wanted to test the underlying technologies and development infrastructure, and to ensure that data is collected, transmitted, stored and retrieved at suitable levels of security of patient identifiable information. Second, we sought feedback on whether patients find it an acceptable aid. Third, we sought feedback on ways to improve the app ahead of a second, larger, deployment.

### App database/data security

Each craving or smoking event logged by the user is sent to a secure server stored within the University of St Andrews School of Medicine. The data are transferred from the phone to the server using Transport Layer Security protocol and industry standard for e-commerce. The data that are collected by the MapMySmoke app at each craving or smoking event are stored on the server after two layers of encryption. We use a double key (private and public) system to store the data in a database, with the outer layer protected by a 2048-bit private key held by the project PI (GH). The data transfer is one-way, i.e. from the phone to the server. Once the encrypted data are on the server, there are two further restrictions regarding use by co-principal investigators: (1) only anonymised data are available; and (2) access to these anonymised data is limited to secure access computer terminals within the School of Medicine at the University of St Andrews.

## Results

**Fig. 2:**
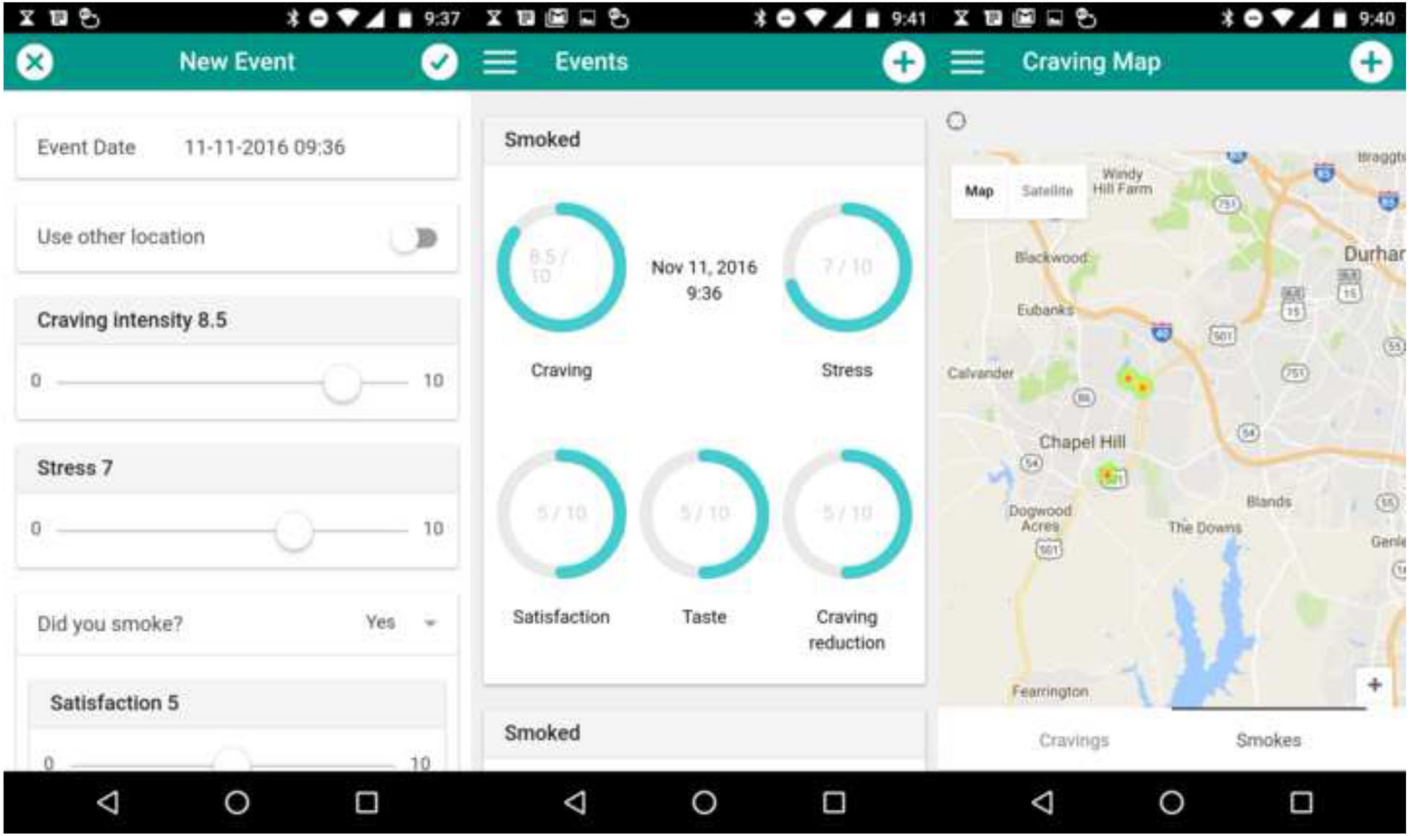
Screenshots from the MapMySmoke app indicating one data entry screen and two visual summaries

**Fig. 3:**
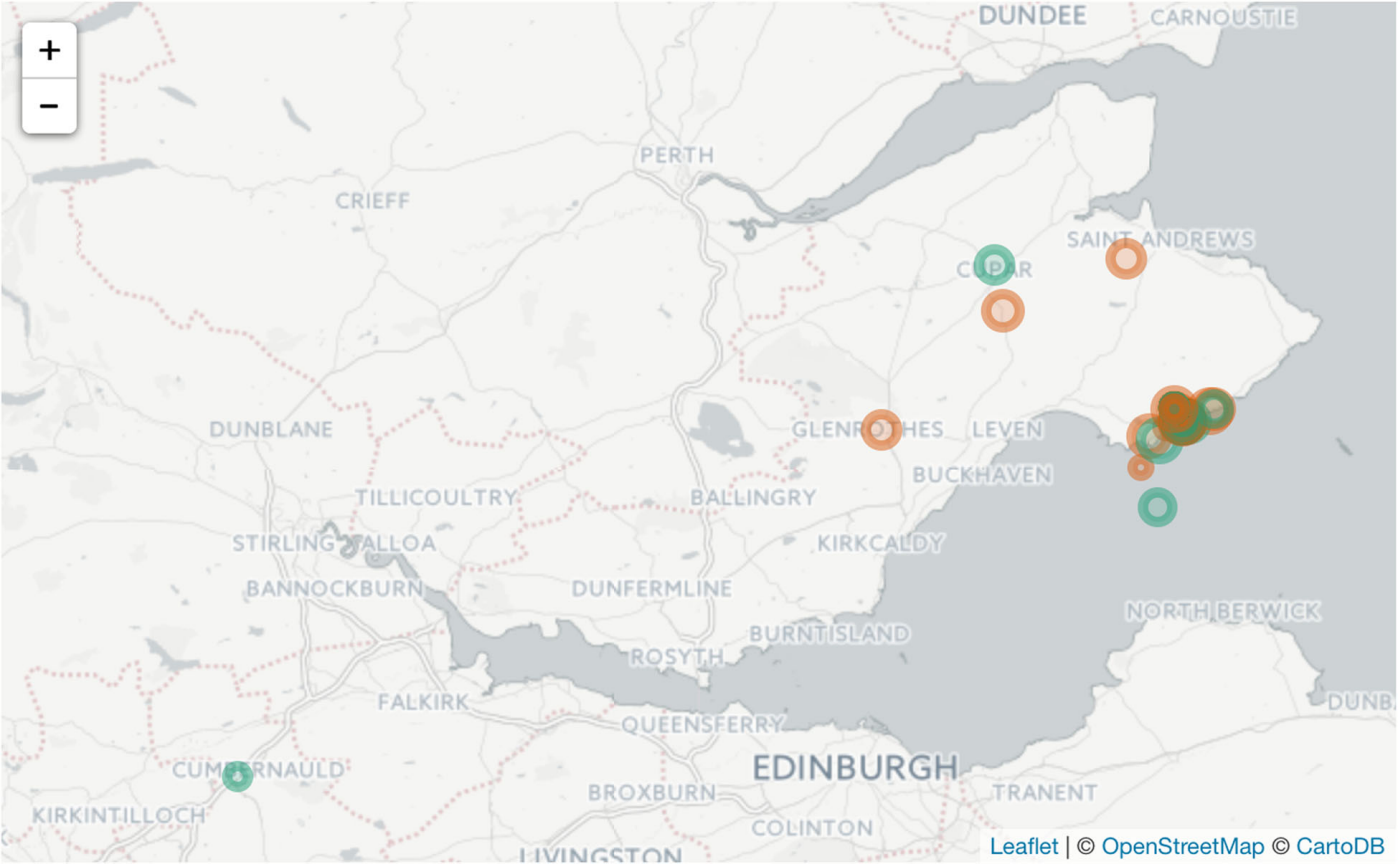
Map showing smoking locations as logged by two users in the first feasibility study. The *colours* indicate the smoker but have been randomised to preserve anonymity. *Size* of the *circle* corresponds to the degree of satisfaction the patient expressed for each smoking event

We consented eight patients in a surgery in Fife and installed the first version of the MapMySmoke app on their phones (Fig. 2). Of these eight, four patients logged regularly (Table 1, Fig. 3). In addition, we obtained direct feedback from three of the patient volunteers—two via face-to-face follow-up interviews and one by phone interview—all three patients completed at least 2 weeks of logging. All three of these patients had been regular smokers for >20 years. Feedback was very positive (Table 2). In particular, patients reported that the act of logging made them more aware and decreased smoking and craving behaviour (Table 2). Two people commented that the logging process reduced the amount they smoke, and one patient was able to quit smoking with the aid of the app and NRT. Two commented that they were surprised at how much they actually smoked having looked at the visual feedback. One commented that it was helpful seeing smoking behaviour; for him, the time and place were helpful in highlighting his smoking habits, which helped him work out a quit smoking strategy. One patient liked receiving the pop-up messages. Another particularly liked the heat map, i.e. the spatial representation of smoking and craving behaviour. A few reported technical difficulties associated with the installation—especially on older phones. Despite repeated follow-up attempts, we did not obtain feedback from all users. We suspect that some of them may have felt guilty about not logging and/or attempting to quit, so they did not report further feedback to the GP. In terms of suggested improvements, one user suggested adding a social component to the app, while another felt the app was simplistic and could use a bit more depth (Table 2). One user felt that they wanted a way to see past comments they had entered. Finally, JM reported that one user suggested implementing a daily or weekly summary of logged smoking behaviour (n.b. this has been implemented for the next version).

**Table 1:** Data collected during the first feasibility trial. Of eight consented patients, four regularly logged. Scores shown are the mean for each of five questions that accompany each smoking event (0–10 scale with 10 being higher, e.g. higher stress ahead of the cigarette and higher craving level)

User	No. of cigarettes	Mean stress score	Mean craving score	Mean taste score	Mean satisfaction score	Mean craving reduction
User 1	14	0.75	5.46	5.57	5.96	5.25
User 2	90	5	4.94	5.08	5.05	5.11
User 3	62	3.775	6.59	3.89	6.29	4.68
User 4	4	4.75	6.25	5	7.37	5.25

**Table 2:** Patient anecdotes reported to GP (J Marston) following initial logging phase. These were in response to specific questions about patient motivation, experience with the app and suggestions for improvement

Patient	Why did you take part in the study?	What did you like about the app?	What would you change about the app?	Do you think the app would help smokers to quit or maintain quitting?
Patient A	Wanted help to quit—failed quit attempts in past	Liked the app as it is. Did not find it difficult to use. Most useful feature was that when a craving was felt, the process of logging diverted her attention and often the craving would subside.	Nothing	Yes. Concerned that when NRT was finished cravings might become a problem. Would definitely consider logging again to maintain quit.
Patient B	Sounded interesting. Multiple failed attempts—allergic to Nicotine patches and ‘did not want to put more chemicals in my body’	Liked the current features and look of the app. Particularly liked that ‘logging’ made you stop and think about having a cigarette rather than smoking being an automatic habit. Logging often delayed having a cigarette	Missed not being able to review previous comments. Thought it would be nice to anonymously look at what other people were saying. Felt that you could gain a lot of support from other quitters. Could there be some sort of anonymised chat room? Could this be part of the app?	Yes—useful for both quitting and maintaining quit. Would like to help with Phase 2.
Patient C	Mixture of wanting to quit and to help medical developments	Quite easy to use—particularly liked watching improvements in life expectancy as he reduced smoking.	Would like to have had more depth to the app—was not able to specify, but thought it was over simplistic.	Yes, but need to be motivated. ‘You get out what you put in’

In terms of the original objectives of the study—determine if we could deploy the app with actual patients in a clinical setting, enable secure logging and transmission of private health information and obtain feedback from patients—we were successful on all counts. While we only had a small number of patients log for an extended period of time, their data were all captured and stored securely; they reported positive feedback after using the app (Table 2) and suggested helpful improvements. Though it was not a goal of the study, all three patients reported that the app helped them reduce or cease their smoking behaviour.

## Discussion

The overarching goals of the MapMySmoke app study, i.e. both the small feasibility study reported here, as well as the larger pilot study, are myriad; the first two are patient focused, while the second two are research focused. First, we wish to facilitate a better understanding of an individual patient’s smoking profile. Similar to a food diary, the goal is to enable users to precisely document where and when they had cigarettes and craving events, and to easily tag each event with quantitative and qualitative metadata. Through the accumulation of data over an initial 2-week logging phase, the app will generate a detailed snapshot of the individual patient’s smoking behaviour.

The second goal is to share these data with the GP or healthcare provider, e.g. medical social worker, to generate an individualised plan to support each patient after they start their quit attempt. The ways in which the app will attempt to do that are as follows:

1. Highlight smoking patterns in time and across space
2. Identify critical cigarettes
3. Identify critical times for craving
4. Pair smoking behaviour with tailored, evidence-based support messages

The third goal—a research goal—is to see if we can identify optimal times and places to send the user dynamic support messages. Ideally, these messages would be sent to an individual’s phone ahead of a likely craving event. As a patient’s craving increases, they are more likely to want to smoke. This shift in desire could be classified as an underlying change in motivational state. That is, some combination of physiological status and psychological triggers could lead to the individual wanting to smoke (note that some of the physiological and psychological aspects can be aided with NRT and/or varenicline; these are routinely offered to patients within NHS Fife and will be offered to any patient participating in the study). One way to quantify the changing probability of shifting into such a smoking state is to fit hidden Markov models to the data logged during the first 2 weeks. If we can quantitatively identify times and places of these shifts, then we can better time the delivery of in-app support messages to the patient. That way, the user can receive a support message when it is most needed by him/her, as opposed to a generic message delivered at the same time each day for every patient. For example, based on their recorded smoking pattern, patient A might be vulnerable to relapse at 3 pm while they are at work, while patient B might be vulnerable to relapse at 1 pm following lunch, etc. If each patient can receive a tailored message in advance of their unique craving event, then we would expect a priori that the intervention would be more relevant. Research has shown that prospective patients are more interested in receiving tailored messages [19].

The fourth goal is to capture the spatial aspects of relapse. This is accomplished simply by tying the logging of a smoking event to the individual’s quit date. The quit date is set at the patient-GP meeting and stored within the app. Should a user log a smoking event after that date, then that first event is stored as a relapse event. We aim to understand the links between the spatial environment and relapse behaviour through investigation of the data associated with post-quit smoking events. In addition to capturing spatial information associated with the relapse, the app will send different messages to the user after a relapse event. Essentially, the structure of the app remains unchanged during this phase, but the context of a smoking event entered post-quit will automatically be tagged as a relapse event. Because this is a different type of event, the next version of the MapMySmoke app will provide the patient with dynamic messages designed to support the user and encourage them to return to the quit attempt. In summary, this goal is both not only research related—in the sense that we will record the spatial aspects of relapse—but also patient focused through the relapse-related messaging.

The results from the feasibility study clearly show that this app can be successfully deployed. Though we caution that it was a very small study limited to one GP practice, we found that patients were comfortable using the app, were able to better understand their smoking behaviour via logging and reported liking the visual feedback from the app. This means that we can use these results and patient-derived feedback (Table 2) to improve the app and test its efficacy on a larger scale. In the larger feasibility study, we will assess efficacy by examining self-reported abstinence rates at 4 weeks, and 3 months following the quit date, and comparing them to baseline data collected by NHS Fife. We chose these dates because they are the dates used by NHS Fife’s Tobacco Cessation Services team, which are in turn following Scottish Government targets.

We will use the corpus of smoking data in at least four unique ways. First, we will use data obtained during the logging phase in an effort to quantify the probability of a user switching into a smoking state. This is of interest from a clinical standpoint, as it helps uncover the myriad determinants of smoking behaviour. Our intention is to use these state-switching probabilities in order to better target support messages to individual patients. To do this, we will use latent-state models in an effort to understand the temporal distribution of smoking behaviour [20]. In later versions of the app, we intend to use these state-switching probabilities as the mechanism for generating targeted ‘just-in-time’ support messages [5] to the patient. Second, we will analyse data obtained during the post-quit phase to quantify if the app-based intervention increases rates of smoking cessation. Third, we will try and fit marked point-process models [21] to the spatial information data recorded at each event. These will be used to better understand the spatial aspects of smoking and craving events across individuals by accounting for the spatial structure inherent in the data. We will also explore fitting temporal marked point-process models to the post-quit data to see if craving maps change over time. Fourth, because the patient records their rate of smoking the first time they encounter the app, we can quantify adherence to using the app by comparing observed smoking rates during the logging phase with those entered by the user at the start of the intervention.

**Fig. 4:**
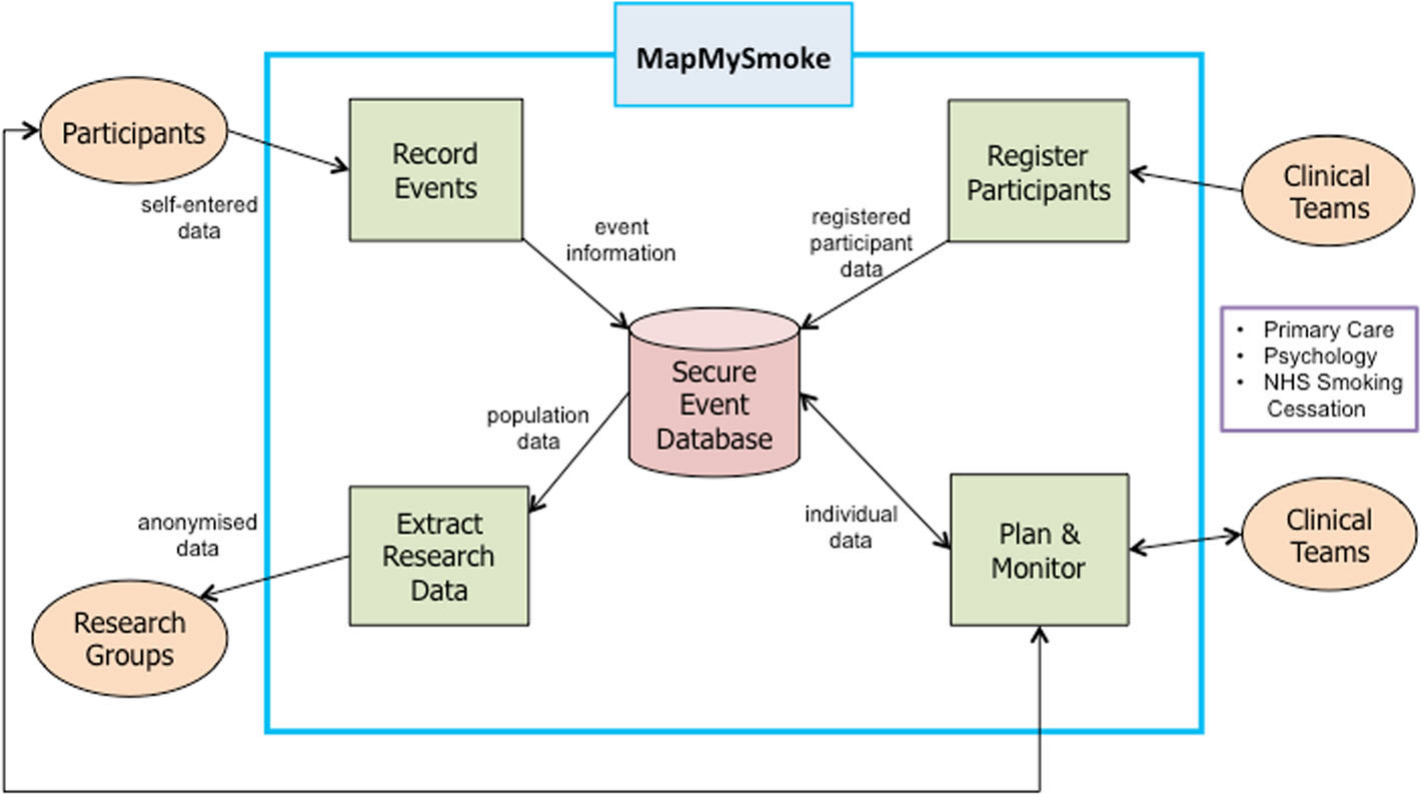
Data flow for the MapMySmoke app

As the project has progressed, we have considered several ways to improve the app. In the apps’ current form, the data encryption and storage scheme does not allow communication from the server back to the phone or to the healthcare provider. Therefore, summary statistics and maps of spatial smoking and craving behaviour are limited to in-app display (Fig. 2). However, in future versions of the schema (Fig. 4), we will offer patients and healthcare providers the ability to receive statistical summaries of the individual behaviour (Fig. 3). This could include initial snapshots of likely behaviour at certain times and places. This may be of great advantage to the GP/healthcare provider in that it could offer them a better understanding of patient behaviour prior to the post-logging interview thereby improving healthcare delivery at the point of care.

In addition, the app could be structured to deliver support to the users at vulnerable times and places. These times and places are unique to each individual, and based on their profiles generated during the logging phase. Prior to implementing this feature, the sampling and communication protocol within the app would be subject to ethical approval within the NHS. While more app-patient interaction and communication is likely to be helpful [19], this must be balanced against data security and privacy concerns [22, 23]. As more lines of communication are made available, data are increasingly vulnerable. In addition, research suggests that the efficacy of sensing must be shown to be reliable and effective before users would be open to the triggering of context-based interventions [19].

Another feature we would like to address in the future is the inclusion of social support with peers. Cheung et al. [8] showed that the inclusion of a social sharing platform (WhatsApp) resulted in fewer relapse events. While the inclusion of such a social feature within MapMySmoke may raise further data security concerns, its possible clinical benefits are worth exploring [19].

In early pre-release versions of the app, we implemented a random forest algorithm [24] to deliver support messages. However, users reported anecdotally that this was reminding them too much about their smoking habit. For example, one user reported that the random forest algorithm was learning that he liked to smoke at home. One day as he walked home, he got a message alerting him to the increased probability of smoking—even though he himself was not craving a cigarette. Thus, the app inadvertently triggered a craving event. Therefore, we need to be careful that increased monitoring and usage of the app has its intended effect—namely aiding smoking cessation—and guard against the triggering or delivery of unwanted smoking cues [19, 22].

Finally, in the first feasibility study, we encountered several candidate patients with older phones that did not support the current version of the MapMySmoke app. This is because we are developing and deploying at the leading edge of mobile technology. However, the remit of the NHS Tobacco Support Services is aimed at populations with higher indices of deprivation. We propose to address this issue of device incompatibility in future versions by developing more backwards compatible versions. While this comes with some added development cost, it opens up the study to more potential users and builds-in equity of health promotion.

## Conclusions

Since more and more humans have and use smartphones, there exists a clear opportunity to harness these tools in the delivery of targeted, specific medical information and support. In particular, we feel there is an opportunity to (a) learn about individual smoking patterns across space and through time and (b) use that information to provide better, more tailored support messages that aid and support a quit attempt. To address this, we have built and successfully deployed a mobile app designed to log individual smoker’s behaviour, provide graphical and statistical summaries of smoking behaviour and support a quit attempt with the delivery of in-app messages designed to provide specific and time-relevant information to the smoker. Initial feedback from users in the first feasibility study was very positive. Thus, the MapMySmoke app could provide practitioners with a new effective tool that supports patients through the difficult process of quitting smoking and maintaining abstinence from smoking.
